# Surgical Training Profiling in Low- and Middle-Income Countries: Peru, Ecuador, and Brazil

**DOI:** 10.7759/cureus.91750

**Published:** 2025-09-06

**Authors:** Vania Arboleda, Aryan Lajevardi, Kawther N Elsouri, Emanuella M Brito, Martina A Maldonado, Maria Fioletova, Pierina S Barletti, Michelle L Demory

**Affiliations:** 1 Department of General Surgery, Cleveland Clinic Akron General, Akron, USA; 2 Department of General Surgery, Hospital Corporation of America (HCA) Florida Orange Park Hospital, Orange Park, USA; 3 Department of Neurology, Corewell Health West/Michigan State University (MSU), Grand Rapids, USA; 4 Dr. Kiran C. Patel College of Osteopathic Medicine, Nova Southeastern University, Davie, USA; 5 Department of Biology, Nova Southeastern University, Davie, USA; 6 Division of Immunology, Dr. Kiran C. Patel College of Allopathic Medicine, Nova Southeastern University, Davie, USA

**Keywords:** general surgery residency training, global surgery partnerships, global surgical access, low- and middle-income countries, medical education, public health, surgical subspecialties

## Abstract

Context

Many people worldwide lack access to surgical care. Surgical access is inequitably distributed worldwide, with the burden falling most heavily on low- and middle-income countries (LMICs) due to geographical, financial, and educational barriers. Due to various challenges, surgical access and training in LMICs differ significantly from high-income countries (HICs). In LMICs, limited resources, infrastructure, and healthcare workforce shortages often lead to inadequate surgical care, with essential surgeries frequently inaccessible to a substantial portion of the population.

Objectives

The goal of this research study is to profile the surgical residents in cities of Peru, Ecuador, and Brazil and highlight the strengths, weaknesses, and limitations of various surgical training programs.

Methods

The research team established a point of contact in each city in its respective country. The researchers, VA, MM, and EB, distributed the emails to Peru, Ecuador, and Brazil's surgical residents. The email contained a link to the survey that asked for non-identifiable information. This research study was designated as "Exempt" by the Nova Southeastern University Institutional Review Board.

Results

Out of the 31 participants emailed, 10 responded and were included in this study. Most respondents are currently in general surgery training programs, and all residents, despite surgical specialty, would like to specialize further. Although they perform classic open surgeries, such as appendectomies and cholecystectomies, these residents express their desire to be trained in laparoscopic procedures. Trainees recognized that their facilities must transfer patients to other, more well-equipped facilities to perform a subset of surgeries. The participants expressed several areas for improvement regarding the outdated procedures and the lack of laparoscopic surgeries. Yet, their programs offer some strengths regarding the faculty and the number of surgeries performed.

Conclusions

Residents from LMICs strongly desire to further subspecialize in their training and continue to create or find opportunities with other LMICs and HICs. Technology, staff, and supply are the main limitations to surgical training in these areas of the world. However, surgical residents from Caruaru, Brazil; Loja, Ecuador; and Lambayeque, Peru, recognize the mentorship and willingness of their faculty as the main strength of their training.

## Introduction

An estimated 4.8 billion people worldwide lack access to surgical care, with the poorest third of the world's population receiving only 3.5% of the world's surgeries [1]. Access to surgical care is essential to healthcare, requiring adequate capacity to perform safe, appropriate, and affordable surgery. Unfortunately, surgical access is inequitably distributed worldwide, with the burden falling most heavily on low- and middle-income countries (LMICs) due to geographical, financial, and educational barriers [2].

Low-income countries (LICs), middle-income countries (MICs), and high-income countries (HICs) are classifications often used by international organizations such as the World Bank to categorize nations based on their gross national income (GNI) per capita [3]. LICs, as defined by the World Bank, are nations with a GNI per capita of $1,045 or less; MICs are those with a GNI per capita between $1,046 and $12,695; and HICs have a GNI per capita of $12,696 or more [3]. These classifications help guide global development policies and resource allocation, with LICs typically receiving more aid and support for poverty alleviation and infrastructure development [4].

HICs are almost three times more effective than LMICs at improving surgical health access and quality, with government expenditure playing a significant role [5]. A leading source of the surgical gap in LMICs is the need for more access to information about surgical care, combined with the low surgical workforce ratio per population [6]. The lack of access is more present in rural areas than urban ones, demonstrating healthcare inequities within the different socioeconomic classes [7]. These social disparities need to be recognized and addressed with a focus on collective action.

Another factor contributing to the limited accessibility in LMICs is the shortage of trained surgeons. In response, many of these countries have developed postgraduate surgical education programs to train surgeons and improve access to surgical care [8]. Some programs improved clinical management to decrease resource use [9]. In contrast, others created global academic partnerships with HICs to enhance the surgical education of residents in LMICs through funding, structured training modules, and increased supervision in the operating room [10,11]. However, with the limited financial resources for healthcare in LMICs and the need for domestic financing of surgical systems, these countries continue to face challenges in access to quality surgical healthcare.

Due to various challenges, surgical access and training in LMICs such as Peru, Brazil, and Ecuador differ significantly from high-income countries (HICs) [12]. In LMICs, limited resources, infrastructure, and healthcare workforce shortages often lead to inadequate surgical care, with essential surgeries frequently inaccessible to a substantial portion of the population [12]. Additionally, the quality of training and educational opportunities for surgical professionals can be suboptimal, potentially affecting the competence and skill of the surgical workforce. Although there is increasing evidence of a lack of proper surgical care in LMICs, it is undeniable that access to safe and effective surgical treatments is a fundamental component of an effective healthcare system [13].

Barriers to surgical training in LMICs prevent the advancement of medicine within their nation and the potential contribution of their surgical advancements to physicians worldwide. Studies have pointed out that interventions to improve surgical care can be cost-effective [12,14]. In a scenario of limited resources, technological improvements can be presented as low-cost solutions to other nations, allowing for more affordable care worldwide [15]. The goal of this research study is to profile the surgical residents in cities of Peru, Ecuador, and Brazil and highlight the strengths, weaknesses, and limitations of various surgical training programs.

## Materials and methods

The research team began data collection on September 1, 2023, by first establishing a point of contact in each city within its respective country. The Peruvian point of contact obtained the emails from the public record of the four existing teaching hospitals in Lambayeque, Peru. The Ecuadorian point of contact obtained the emails from the academic office of the teaching hospital in Loja, Ecuador. The Brazilian point of contact obtained the emails from the academic office of the teaching hospital in Caruaru, Brazil. The researchers, VA, MM, and EB, distributed the emails to the respective points of contact in Peru, Ecuador, and Brazil, which were then shared with other surgical residents. The email contained a link to a Google Forms survey that asked for non-identifiable information. The survey was translated into Spanish before distribution in Peru and Ecuador and into Portuguese before distribution in Brazil. The English version of the questionnaire is attached in the Appendices.

The survey was developed to evaluate the demographic characteristics, clinical workload, surgical exposure, and perceptions of training strengths and limitations among surgical residents. It consisted of 20 questions, including multiple-choice, checkbox, Likert-type, and open-ended questions. Input from medical and surgical educators was additionally obtained to ensure relevance for the training environment in Peru, Ecuador, and Brazil. A pilot trial was conducted with five medical students to assess the clarity, ease of understanding, and completion time of the survey. The students were also asked to identify any confusing language. The focus of the pilot trial was to evaluate survey structure and usability. Based on the feedback from the medical educators and medical students, the team made modifications to the wording of questions and answer options.

The participants eligible for selection were those actively enrolled in a surgical residency program, including general surgery, orthopedics, ophthalmology, neurosurgery, obstetrics and gynecology, and otorhinolaryngology at the time of the study. To be included in the study, the surgical residency program had to be in an LMIC. Residents in HICs, medical students, attending surgeons, or individuals not enrolled in a surgical residency program were excluded from the study. Participation was voluntary, and we did not collect any personal identifying information to maintain participant confidentiality. This research study was designated as "Exempt" by the Nova Southeastern University Institutional Review Board. Data collection officially ended on December 15, 2023.

## Results

Out of the 31 participants who were emailed, this research team received 10 responses, with five participants from Peru, two from Ecuador, and three from Brazil.

**Figure 1:**
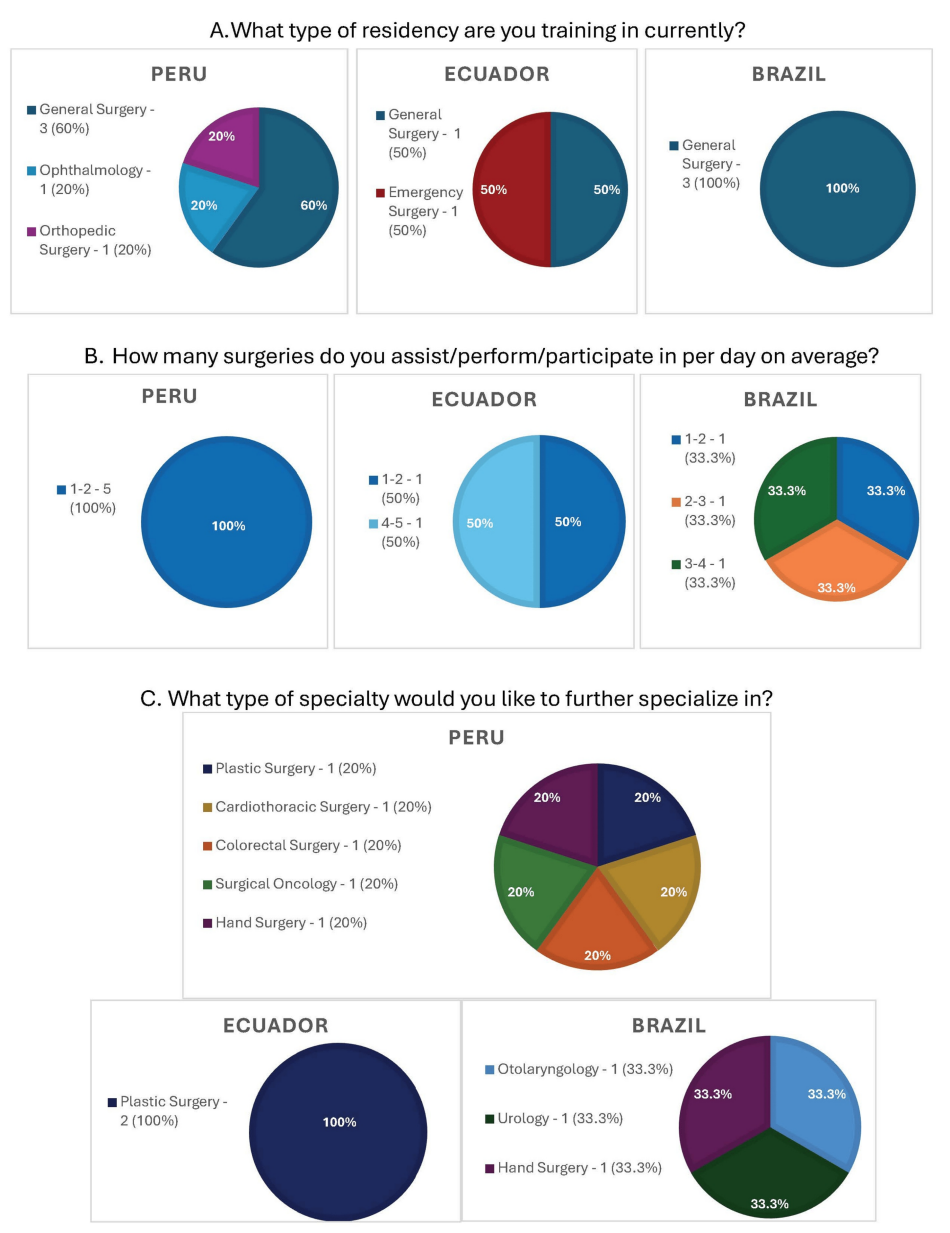
Profile of surgical specialties by country (A). Number of surgeries performed daily by country (B). Desired fellowships by country (C). Out of the 10 participants, five were from Peru, two were from Ecuador, and three were from Brazil. Note: Answers with 0% response rate were not outlined in the figure.

In the state of Lambayeque, Peru, 60% of the participants answered that they were in a general surgery residency, 20% in an orthopedic surgery residency, and 20% in an ophthalmology surgery residency. In the state of Loja, Ecuador, 50% of the participants answered that they were in a general surgery residency and 50% in an emergency surgical residency. In the state of Caruaru, Brazil, 100% of the participants answered that they were in a general surgery residency (Figure 1A). All participants from Peru reported performing 1-2 surgeries per day. Fifty percent of Ecuadorian participants reported performing 1-2 surgeries per day, while the other 50% reported performing 4-5 surgeries per day. Of the Brazilian participants, 33.3% reported performing 1-2 surgeries per day, 33.3% reported performing 2-3 surgeries per day, and 33.3% reported performing 4-5 surgeries per day (Figure 1B). Peruvian trainees reported future interest in cardiothoracic surgery, hand surgery, plastic surgery, colorectal surgery, and oculoplastics. All participants in Ecuador indicated their interest in further specializing in plastic surgery, while Brazilian trainees demonstrated an equal interest in hand surgery, otolaryngology, and urology (Figure 1C).

Peruvian general surgery trainees reported appendectomy and open cholecystectomy as the most performed surgeries during their training, while Peruvian orthopedic and ophthalmologic trainees reported osteosynthesis of the lower extremity and pterygium surgery as the most commonly performed surgeries, respectively. Both Ecuadorian trainees reported appendectomy as the most commonly performed surgery, while Brazilian trainees reported appendectomy and laparoscopic cholecystectomy (LC) as the most commonly performed surgeries (Table 1). Peruvian general surgery trainees reported their desire to gain training in laparoscopic hernioplasty, open hernioplasty, and abdominal wall repair, while Peruvian orthopedic and ophthalmologic trainees expressed a desire for arthroplasty and cataract training, respectively. Ecuadorian trainees reported their desire to train in face transplant and open colectomy, while Brazilian trainees reported their desire to train in laparoscopic hernioplasty and open cholecystectomy (Table 1). Additionally, the main reason for transfers in all three countries was due to the lack of technology. Other reasons identified by the trainees include the lack of logistics, intensive care units (ICUs), operating staff, nutritional support, and academic formation (Table 1).

**Table 1:** Most performed surgeries, surgeries wished to be trained in, commonly transferred surgeries, and reasons for the surgical transfer separated by country. Note: Numbers in parentheses indicate total responses from surgical residents. Each resident was allowed to give more than one response.

	Peru	Ecuador	Brazil
Most Performed Surgeries	Appendectomy (2)	Appendectomy (2)	Appendectomy (2)
	Open Cholecystectomy (2)		
	Osteosynthesis of Lower Extremity (1)		Laparoscopic Cholecystectomy (2)
	Pterygium Surgery (1)		
Surgeries Wished to be Trained In	Laparoscopic Hernioplasty (2)	Face Transplant (1)	Laparoscopic Hernioplasty (2)
	Open Hernioplasty (1)		
	Abdominal Wall Repair (1)	Open Colectomy (1)	Open Cholecystectomy (1)
	Arthroplasty (1)		
	Cataract Surgery (1)		
Most Commonly Transferred Surgeries	Exploratory Laparotomy (2)	Spinal Surgery (1)	Gastroduodenal Pancreatic Surgery (1)
	Short Bowel Syndrome Surgery (1)		Obstetrics (1)
	Pelvic Osteosynthesis (1)	Neurosurgery (1)	Hepatectomy (1)
	Retina Surgery (1)		
Reason for Transfer	Lack of Technology/Machines (5)	Lack of Technology/Machines (2)	Lack of Logistics (2)
	Lack of Logistics (2)		
	No Intensive Care Unit (1)		Lack of Operating Room Staff (1)
	No Operating Room Staff (1)		
	Lack of Nutritional Support (1)		Lack of Technology/Machines (1)
	Lack of Academic Information (1)		

## Discussion

Profile of residents, subspecialty, and training

This study outlines the involvement of residents in general surgery, orthopedic surgery, and ophthalmology in three LMICs: Peru, Brazil, and Ecuador. All general surgery residents from the three countries examined in this study reported a desire to further subspecialize following their training. Medical subspecialization is accelerating in LMICs as more residency programs are being established, such as the four-year otolaryngology program in Quito, Ecuador, that also recently introduced a novel facial plastic and reconstructive surgery teaching module with didactics, surgical simulations, and live training [16]. This novelty has increased the interest of other Ecuadorian residents in the subspecialty, as shown in our results with the surgical residents in Loja. Increasing the number of trained surgeons in these countries will help diminish the discrepancy between surgical demand and capacity, reducing fatality and improving overall health outcomes [17-20].

Global surgery is constantly evolving to provide equitable surgical care and training worldwide [21]. Some LMICs offer fellowships in specific subspecialties, such as the CURE Hydrocephalus and Spina Bifida fellowship in Uganda, which has trained surgeons from 20 LMICs to equip them further with state-of-the-art surgical skills to manage childhood neurological conditions [20]. Laparoscopic cholecystectomy (LC) was introduced to Nigeria in 2008, with only up to 31 reported cases performed by 2015 [22]. However, in our study, some residents from Brazil perform LC, those in Peru perform solely open cholecystectomies, and most residents in all three LMICs expressed their desire to gain training in laparoscopic procedures. Although a lack of funding and resources can prohibit specific surgical rotations for residents, collaborations with academic hospitals in HICs have significantly impacted the way residents in LMICs receive their training [23-26]. One program, launched in 2012, deployed about 100 American faculty members annually to academic and clinical teaching settings in Rwanda to train physicians in specialty and subspecialty areas, including orthopedics, neurosurgery, obstetrics and gynecology, and otorhinolaryngology. These collaborations have greatly aided global training efforts [27].

Surgeries performed, transferred, and overall volume

The volume of surgeries performed in LMICs and HICs depends on the scale of surgical services provided by the hospitals in these countries [1]. The Lancet Commission on Global Surgery established a surgical rate of 5,000 procedures per 100,000 individuals as the minimum standard to be achieved by each country, with most LMICs failing to reach this goal [1]. Regarding Latin America, both Peru and Brazil fall below this standard with a rate of 1,174 and 4,433 procedures per 100,000 individuals, respectively [28,29]. These national averages reflect our results, with trainees from Brazil having the largest volume of surgeries performed by residents in a day (4-5 procedures) compared to Peru, which has the lowest volume (1-2 procedures). On the other hand, HICs easily surpassed this goal. For instance, New Zealand, the country with the lowest national surgery rate, has an average of 6,270 surgical procedures per 100,000 people [30].

Although surgical availability in LMICs has begun to increase over the years, there is still a significant disparity in the surgical procedures performed. The most frequently performed surgeries in LMICs include cesarean sections, hernia repairs, appendectomies, cataract surgeries, and fracture surgeries [31-34]. Our results follow this trend as the general surgery residents reported appendectomies as the most highly performed surgery, and the orthopedic residents reported fractures as the primary surgery. Yet, the ophthalmology resident stated that pterygium surgery was the most conducted in comparison to the trend of cataract surgery. These most common procedures significantly contribute to the specific surgical needs in LMICs, dictating the specialties in which most residents are involved. Thus, there are more severe deficiencies in the capacity of surgical oncology, neurosurgery, and pediatric surgery in LMIC, which have the capacity to rapidly evolve as training expands [35-38]. However, transferring surgical cases to other facilities may sometimes be necessary to increase the efficiency of care and optimize surgical capacity.

Our findings identify exploratory laparotomy and gastrointestinal surgeries as the most transferred, followed by pelvic, spinal, and neurological surgeries. Reasons for these transfers include the lack of technology and equipment, intensive care units, operating room staff, and academic training. Although very few studies have examined the prevalence of interhospital transfers in LMICs, Truche et al. reported that the most common surgical conditions that required transfer in Cali, Colombia, were fractures, appendicitis, wounds, abdominal pain, trauma, and acute abdomen. Surgical cases accounted for about 30% of the transfers executed due to limited surgical capacity at the arriving facility [39]. Surgical transfers are also commonly seen in HICs, particularly in rural settings with smaller facilities [40]. In the United States, almost two million interhospital transfers occur yearly [41]. Most frequently, these involve nontraumatic surgical emergencies that require surgical subspecialist expertise to intervene [41,42]. Although patient transfer is associated with delayed care, the increased length of hospital stay, and overall cost, leading to secondary over-triage [39,43,44], motivation for transfers in both HICs and LMICs stems from the need for a higher level of care or specialized services. These are incredibly beneficial to optimize patient care.

Strengths and limitations in training

Currently, of the 300 million surgical procedures performed annually worldwide, only 6% of these are done in the poorest countries, which inhabit one-third of the world's population, and an additional 140 million procedures are needed to reduce mortality in these countries [1]. This unmet need for surgical care in LMICs is attributable to costs for adequate medical supplies and equipment, limited surgical capacity and subspecialty training, and the lack of healthcare facilities. The most reported weakness by the participants was the lack of modern laparoscopic surgeries performed. Others included limited operating room availability, the lack of research, and few video training learning opportunities. Thus, many surgical residents' training in LMICs may be limited to where they can complete surgical training and are forced to travel abroad to receive specialized training [45].

Global surgery fellowships have grown with increasing international collaborations between LMICs and HICs. The duration of these fellowships varies, most lasting between one and two years, after which trainees return to their home country after completion [46]. Although only one-quarter of these fellowships were open solely to LMIC trainees, they provide these surgeons with funded and mentored opportunities to further pursue subspecialty training and return home with their newly learned skills [46]. Other programs involve a one-year pediatric surgical training experience between Canada and Uganda [47]. Surgical residents in HICs have also displayed an increasing interest in completing electives in LMICs, which can further help train surgeons in these developing, resource-limited areas through mentorships with HIC-trained surgeons [17,18]. Interest in orthopedic surgery in LMICs is also growing, and many North American orthopedic surgery residency programs offer international training in these LMICs that can further aid resident training [48]. Over the last few decades, there have been efforts to alleviate this burden by implementing partnerships with academic and community-based programs in HICs. However, some curriculum differences might limit this interaction [21].

The most considerable distinctions in training curricula between LMICs and HICs were reported to be the lack of relative standards to guide education, the need to ensure the curricula, the inconsistent supervision and bedside teaching, and inadequate access to clinical and educational resources [49]. These coincide with the weaknesses outlined by some residents in our study, including a lack of research and video training learning opportunities. Wilkinson et al. also identify the lack of experienced trainers as one of the most consequential barriers to advanced surgical education [15]. In LMICs, residents are usually relied on as the primary surgeons. Yet, surgical faculty often need to be more involved, which poses the question of how effectively surgical residents are being trained [19]. Nonetheless, the willingness of the faculty to teach, the complex cases, and the sheer volume of surgeries performed, allowing for greater surgical practice, were reported as strengths in these surgical programs. As a result, a continuation of LMIC-HIC partnerships and more recent online electronic learning models for surgical teaching will be necessary to help expand training programs in LMICs.

In summary, most respondents are currently in general surgery training programs, and all residents, despite surgical specialty, would like to specialize further. Although they perform classic open surgeries, such as appendectomies and cholecystectomies, these residents express their desire to be trained in laparoscopic procedures. Trainees recognized that their facilities must transfer patients to other, better-equipped facilities to perform a subset of surgeries. The participants expressed several areas for improvement regarding the outdated procedures and the lack of laparoscopic surgeries. Yet, their programs offer some strengths, particularly in terms of faculty and the number of surgeries performed.

Limitations of the study

This study had several limitations that were considered when interpreting the findings. To start, the study's low number of surgical residents participating and the limited number of participants from each country were limitations. Ideally, we would have wanted more responses from each country to ensure well-rounded responses; however, the responses received effectively represent the strengths and weaknesses of surgical training in LMICs. Also, medical students conducted the pilot test for questionnaire clarity and usability, rather than surgical residents. Although the pilot's purpose was restricted to evaluating language clarity and survey logistics, this population difference may have limited ambiguities specific to the context of surgical residency. Additionally, although the survey was translated by medical students whose native language was Spanish and Portuguese, professional or certified interpreters were not used. We would like to add that the survey relied on self-reported data, which may be subject to recall bias, social desirability bias, or personal interpretation, particularly in questions regarding surgical caseload, perceived training quality, and future career interests. These subjective measures may not accurately reflect objective clinical exposure or program characteristics. Lastly, the cross-sectional design captured the residents' perspectives at a single point in time, which may not have reflected the changes in workload, training opportunities, or program limitations that occur throughout a residency program. Despite these limitations, this study provides valuable preliminary insights into the demographic profiles, surgical exposure, training challenges, and aspirations of surgical residents in Peru, Ecuador, and Brazil. This study can serve as a foundation for future, more comprehensive evaluations of surgical training programs in the region.

## Conclusions

Surgical training continues to develop in low- and middle-income countries with national pipeline programs and aid from high-income countries. Residents from LMICs strongly desire to further subspecialize in their training and continue to create or find opportunities with other LMICs and HICs. Technology, staff, and supplies are the main limitations to surgical training in these areas of the world. However, surgical residents from Caruaru, Brazil; Loja, Ecuador; and Lambayeque, Peru, recognize the mentorship and willingness of their faculty as the main strength of their training.

## Appendices

English version of the questionnaire

*Required 

1. Age* (Check only one option)

· 18-25

· 26-30

· 31-35

· 36-40

· Over 40

2. Gender* (Check only one option)

· Female

· Male

3. Are you a Brazilian citizen?* (Check only one option)

· Yes

· No

4. Are you a resident of the state of Pernambuco, Brazil?* (Check only one option)

· Yes

· No

5. In which city did you study medicine?* (Check only one option)

· Caruaru

· Recife

· Other:

6. What type of surgical residency are you currently doing?* (Check only one option)

· General Surgery

· Gynecology and Obstetrics

· Anesthesiology

· Ophthalmology

· Orthopedic Surgery

· Other:

7. What year of residency are you currently in?* (Check only one option)

· R1

· R2

· R3

· R4

· Other:

8. How many surgeries do you attend/perform/participate in on average per day?* (Check only one option)

· 1-2

· 2-3

· 4-5

· More than five

· More than 10

9. Are you interested in pursuing an additional subspecialty after residency?* (Check only one option)

· Yes

· No

10. What type of subspecialty would you like to pursue?* (Check only one option)

· Plastic Surgery

· Cardiothoracic Surgery

· Colorectal Surgery

· Oncologic Surgery

· ENT (Otorhinolaryngology)

· Oculoplastics

· Neurosurgery

· Hand Surgery

· Fetal Medicine

· Other:

11. How many hospitals do you rotate through per year?* (Check only one option)

· 1

· 2

· 3

· 4

· More than five

12. How many operating rooms are there in your hospital?* (Check only one option)

· 1

· 2

· 3

· 4

· More than five

13. On average, how many hours per day do you spend in the operating rooms?* (Check only one option)

· 5-8

· 8-10

· 10-12

· 12-15

· More than 15

14. What is the most common surgery that you attend/perform/participate in?*

15. Which surgery would you like to receive training in but are currently unable to perform due to your level of training?*

16. What is the most common type of surgery that you refer to another hospital?*

17. What is the reason for referring this type of surgery to another hospital?* (Check all that apply)

· Lack of technology/equipment

· Lack of training

· Lack of staff

· Scheduling/logistics issues

· Other:

18. What are the weaknesses of the surgical training you are receiving?*

19. What are the strengths of the surgical training you are receiving?*

20. What is your English proficiency level?* (Check only one option)

· No proficiency

· 1

· 2

· 3

· 4

· 5

· Fluent
